# Influence of Intrinsic Aerobic Exercise Capacity and Sex on Cardiac Injury Following Acute Myocardial Ischemia and Reperfusion

**DOI:** 10.3389/fcvm.2021.751864

**Published:** 2021-11-26

**Authors:** Musaad B. Alsahly, Madaniah O. Zakari, Lauren G. Koch, Steven Britton, Laxmansa C. Katwa, Robert M. Lust

**Affiliations:** ^1^Department of Physiology, Brody School of Medicine, East Carolina University, Greenville, NC, United States; ^2^Department of Physiology, College of Medicine, King Saud University, Riyadh, Saudi Arabia; ^3^Department of Physiology, College of Medicine, Taibah University, Medina, Saudi Arabia; ^4^Department of Physiology and Pharmacology, University of Toledo, Toledo, OH, United States; ^5^Departments of Anesthesiology and Molecular and Integrative Medicine, University of Michigan, Ann Arbor, MI, United States; ^6^East Carolina Diabetes and Obesity Center, East Carolina University, Greenville, NC, United States

**Keywords:** aerobic capacity, HCR, LCR, intrinsic, gender, coronary occlusion

## Abstract

**Purpose:** Previous reports have suggested that active exercise aside, intrinsic aerobic running capacity (Low = LCR, high = HCR) in otherwise sedentary animals may influence several cardiovascular health-related indicators. Relative to the HCR phenotype, the LCR phenotype is characterized by decreased endothelial reactivity, increased susceptibility to reperfusion-induced arrhythmias following short, non-infarction ischemia, and increased diet-induced insulin resistance. More broadly, the LCR phenotype has come to be characterized as a “disease prone” model, with the HCRs as “disease resistant.” Whether these effects extend to injury outcomes in an overt infarction or whether the effects are gender specific is not known. This study was designed to determine whether HCR/LCR phenotypic differences would be evident in injury responses to acute myocardial ischemia-reperfusion injury (AIR), measured as infarct size and to determine whether sex differences in infarction size were preserved with phenotypic selection.

**Methods:** Regional myocardial AIR was induced *in vivo* by either 15 or 30 min ligation of the left anterior descending coronary artery, followed by 2 h of reperfusion. Global ischemia was induced in isolated hearts *ex vivo* using a Langendorff perfusion system and cessation of perfusion for either 15 or 30 min followed by 2 h of reperfusion. Infarct size was determined using 2, 3, 5–triphenyltetrazolium chloride (TTC) staining, and normalized to area at risk in the regional model, or whole heart in the global model. Portions of the tissue were paraffin embedded for H&E staining and histology analysis.

**Results:** Phenotype dependent differences in infarct size were seen with 15 min occlusion/2 h reperfusion (LCR > HCR, *p* < 0.05) in both regional and global models. In both models, longer occlusion times (30 min/2 h) produced significantly larger infarctions in both phenotypes, but phenotypic differences were no longer present (LCR vs. HCR, *p* = n.s.). Sex differences in infarct size were present in each phenotype (LCR male > LCR female, *p* < 0.05; HCR male > HCR female, *p* < 0.05 regardless of length of occlusion, or ischemia model.

**Conclusions:** There is cardioprotection afforded by high intrinsic aerobic capacity, but it is not infinite/continuous, and may be overcome with sufficient injury burden. Phenotypic selection based on endurance running capacity preserved sex differences in response to both short and longer term coronary occlusive challenges. Outcomes could not be associated with differences in system characteristics such as circulating inflammatory mediators or autonomic nervous system influences, as similar phenotypic injury patterns were seen *in vivo*, and in isolated crystalloid perfused heart *ex vivo*.

## Introduction

Enhanced aerobic capacity has been associated with diminished morbidity, improved quality of life, and decrease risk for cardiovascular diseases (1–3). Coronary artery disease is currently the leading cause of mortality and morbidity in the western world. The most common fatal consequence of coronary artery disease is an acute myocardial infarction, and death due to coronary artery disease appears to decrease with treatments that include an aerobic exercise component (2, 3). It remains unclear whether the benefits of exercise derive primarily from altered risk profiles (lipid, obesity, etc.) or directly from intrinsic myocardial resistance to injury.

As understanding of the mechanisms involved with tissue injury following arterial occlusion (ischemia), and then subsequent appreciation of additional injury associated with relieving the occlusion (reperfusion) advanced (4–6), it became clear that periods of intermittent ischemia that either immediately preceded the ischemic event (ischemic preconditioning), or immediately preceded the reperfusion event (ischemic post-conditioning) could induce multiple pathways that could limit the extent of injury and improve recovery (4–7). Pre-and post-conditioning effects were associated with erythropoietin, the heme-oxygenase system, and atrial natriuretic peptide among many others (8–10), and those effects could I turn depend on underlying conditions such as diabetes, hyperlipidemia, and estrogen status (8–10). Practically though, both pre-conditioning and post-conditioning have had limited success, simply due to the inherent challenges of creating controlled, intentional ischemia. Over the last 15 years, the phenomenon of remote preconditioning has been introduced, where mild demand ischemic challenges in distal regions, such ands an arm or leg, would confer protection against subsequent ischemia in critical organs, such as heart (4). Active exercise increasingly has become the option of choice both for reducing the impact of underlying conditions/comorbidities, as well as upregulating processes that could be recruited by pharmacologically and mechanically induced protective strategies (4, 5).

Aerobic exercise capacity has genetic and environmental components. The genetic component define the intrinsic capacity for endurance exercise and appears to have two parts, the genes that regulate adaptive responses to active exercise training and the genes that determine intrinsic exercise capacity, regardless of actual activity (11–14). Although aerobic exercise training has beneficial effects on several of cardiovascular diseases, the variability in the physiological response to exercise training suggests potential impact of the genetic composition. It has been estimated that up to 60–70% of the variation in exercise capacity is due to the genetic component (11, 13, 14). It is not clear if the genetic component for enhanced exercise capacity alone can result in protection from cardiovascular diseases or whether the training stimulus is necessary to produce the positive results, or if a combination of both is required. The development of a novel rat model using treadmill based phenotypic selection as a basis for breeding rather than specific gene manipulation has advanced the ability to differentiate the genetic components from the environmental effects, such as exercise training, that also influence the aerobic capacity (15–17). This novel rat model contrasts intrinsic aerobic capacity as a phenotype, low-capacity runners (LCR) and high-capacity runners (HCR), provides a tool to experimentally address the intrinsic component of exercise and its contribution to cardiovascular disease (15). Untrained low endurance running capacity (LCR) rats were found to have a higher incidence of risk factors associated with cardiovascular disease than their untrained high endurance running capacity (HCR) counterparts (15, 18). The LCR rats were more insulin resistant, had higher mean blood pressures, decreased nitric oxide mediated vascular relaxation, and had lower expression of proteins critical to skeletal muscle fatty acid oxidation (15, 18). LCR animals were predisposed to weight gain and increased blood free fatty acid (FFA) levels compared to HCR counterparts when challenged with a high fat diet (18). Moreover, compared to the HCRs, the LCRs displayed higher arrhythmogenicity following short term myocardial ischemia and reperfusion (13).

Early studies in this model also demonstrated differences in several skeletal muscle metabolic and vascular endpoints. Early studies indicated that HCR rats demonstrated increased VO_2max_ that was attributed to an increased O_2_ capacity and/or increased capillary density in skeletal muscle under both normoxic and hypoxic exercise conditions (19–21). Later studies also indicated that HCR rats had increased VO_2max_ while LCRs appeared to have decreased VO_2max_ values. In addition to increased skeletal muscle capillary density and increased oxidative enzymes reported previously, HCRs now also were demonstrating higher maximum cardiac stroke volume (SV) values in comparison to their LCR counterparts (20).

Noland et al. demonstrated that the LCR phenotype gained more weight and lost insulin sensitivity on a high fat diet compared to HCR (18). Results also have suggested that artificial selection for endurance running capacity selects for intracellular pathways that provide protection against metabolic and cardiovascular stresses. At the cellular level, it has been demonstrated that HCRs show larger amplitude calcium transients and higher efficiency in energy production (19), along with faster skeletal sarcomeric shortening and relaxation (20, 22). Still, cardiac and peripheral muscle metabolism are quite different, as are collateral circulation patterns, insulin sensitivity and responses to ischemia. Despite suggestions that might suggest a cardioprotective effect of intrinsic aerobic capacity, it is not clear whether such an effect actually is present, or whether the known exercise benefit in cardiovascular disease management is limited to active exercise induced effects. While the breeding selection process was initiated and developed with equal cohorts of males and females, the great majority of experimental studies using the model have been in male offspring. There are well-know differences in cardiac ischemia outcomes between males and females (23, 24), and it is not clear whether an intrinsic exercise capacity effect on cardiac ischemia, if present, would be evident in both male and female HCR and LCR rats.

## Materials and Methods

### Animal Strain

The development of LCR and HCR rats has been described previously in detail (16, 25–27). Rats were selected from a heterogeneous rat population in the N:NIH stock (National Institutes of Health, USA) based on inherent running capacity. Endurance running capacity was assessed at 11 weeks of age using run time and distance to exhaustion on a treadmill as parameters. The highest 20% in running capacity of each gender were randomly inbred/backbred to produce the HCR strain and the lowest 20% in running capacity were inbred/backbred to produce the LCR strain. Subsequent generations were assessed and bred in a similar fashion with precautions taken to minimize inbreeding (<1% per generation). Both female and male HRC and LCR rats, 16–18 months of age, from generation 17 were used in this investigation. There were a total of 8 groups: 4 groups (HCR male, HCR female, LCR male, LCR female, *n* = 12 each) underwent regional ischemia, and 4 groups (HCR male, HCR female, LCR male, LCR female, *n* = 12 each) underwent global ischemia. Half of each group (*n* = 6) went through either the 15 min or the 30 min ischemia challenge, followed by 2 h of reperfusion in all animals. Until study, all animals were maintained in constant temperature environments (22°C) with 12/12 light dark cycles, and *ad libitum* access to water and food (standard rat chow, Research Diets, New Brunswick NJ, USA). All animal procedures were approved by the East Carolina University Institutional Animal Care and Use Committee and conformed to the standards in the National Institutes of Health (NIH) Guide for the Care and Use of Laboratory Animals.

### Regional Acute I/R Injury

Regional AIR was induced using procedures essentially as described previously (28, 29). Briefly, all animals were anesthetized (Ketamine/Xylazine) and mechanically ventilated with room air. The heart was exposed through a thoracotomy performed in the left fifth intercostal space. The pericardium was gently separated, and a ligation of the left anterior descending coronary artery was performed using 6.0 suture and a reversible snare. Occlusion was confirmed by cyanosis of the distal myocardial tissue. Following 15- or 30-min occlusion, the snare was released, and the tissue was reperfusion for 2 h. There is a high risk of lethal arrhythmia in this occlusion model, and the availability of animals was limited enough that expected mortality could have compromised the reliability of the infract size measurements, a primary endpoint. To limit the risk of arrhythmia and optimize successful completion of reperfusion, i.p. lidocaine was administered as prophylaxis 5 min before coronary occlusion, 5 min before reperfusion, and 15 min following reperfusion. Similarly, we avoided instrumenting the heart or manipulating the ventricle to the greatest extent possible so as to maintain the number of animals successfully completing the full IR protocol. The approach did work, but it also meant that hemodynamics and assessment of arrhythmia were not completed in the regional IR animals.

### Global Acute Ischemia/Reperfusion Injury

Global AIR was induced using procedures essentially as described previously (30, 31). Briefly, after anesthesia was induced using (Ketamine/Xylazine), the thorax was opened and the beating heart was excised rapidly. The aorta was cannulated, the heart was mounted in a constant pressure, Langendorff system, and perfusion was immediately initiated using Krebs-Henseleit buffer (composition (mM): NaCl 118; KCl 4.7; MgSO_4_ 1.2; KH_2_PO_4_ 1.2; NaHCO_3_ 25; CaCl_2_ 1.4; glucose 11; pH 7.3–7.4) aerated with 95% O_2_/5% CO_2_. System and buffer temperatures were maintained at 37° continuously. Perfusion pressure was set to 80 mm Hg by reservoir height and verified by direct pressure sampling via stopcock at the level of the infusion cannula. Constant preload was established by placing an LV balloon and inflating to a diastolic pressure of 5 mm Hg. The hearts were not paced and were permitted to beat spontaneously. Electrocardiogram and LV pressures were obtained in these animals for subsequent hemodynamic and rhythm assessments. Rhythms were graded on an 9 point scale, ranging from 0, equating to fewer than 50 isolated premature ventricular contractions (PVCs) up to a score of 8, which equated to non-reverting (lethal) ventricular fibrillation within 1 min following reperfusion (30).

### Determination and Quantification of Infarct Size

TTC staining is a well-established procedure for identifying infarct size (28–31). Following reperfusion in the regional model, and while continuously anesthetized, the animals were euthanized by rapid excision of the heart. The aorta was cannulated and the heart was infused retrogradely with a 1.0% solution containing 2,3,5-triphenyltetrazolium chloride (TTC) to delineate the infarcted area. The coronary artery was re-occluded at the original ligature site and a 1.0% solution containing methylene blue was infused through the aortic cannula as counterstain to delineate the area at risk, followed by transverse sectioning of the entire heart. In the global ischemia model, the heart was removed from the apparatus and transverse sectioning was completed immediately. The sections were transferred to a shaker bath containing a 1% TTC solution and incubated for 5 min at 37°C. Once stained, all sections were photographed form both sides using a digital camera. From these photographs, the LV area, area at risk, and area of infarction in each image were determined using NIH image software (ImageJ, version 1.34s). In the regional model, the area at risk was expressed as a percentage of the LV area and the area of the infarcted zone was expressed as a percentage of the area at risk. In the global model, since the entire ventricle was “at risk,” the infraction was expressed as % of whole heart.

### Histology

Histology was performed on the same tissue sections used for infarct measurements. Following TTC staining and digital photography, one of the myocardial sections was fixed in 4% paraformaldehyde, embedded in paraffin wax, and cut at 5-um sections. Sections were stained with hematoxylin and eosin (H&E) and examined by light microscopy under high magnification (X 60). The number of infiltrated neutrophils in each high–power field was counted and normalized to the area of the field, based on the microscope specifications. Neutrophil counts were made on five sections per rat heart and on five fields per section.

### Statistics

Differences between groups were determined by ANOVA and Tukey's *post hoc* test with significance determined when *p* < 0.05. Data in all figure are expressed as mean ± SD.

## Results

### Acute I/R Injury

**Figure 1:**
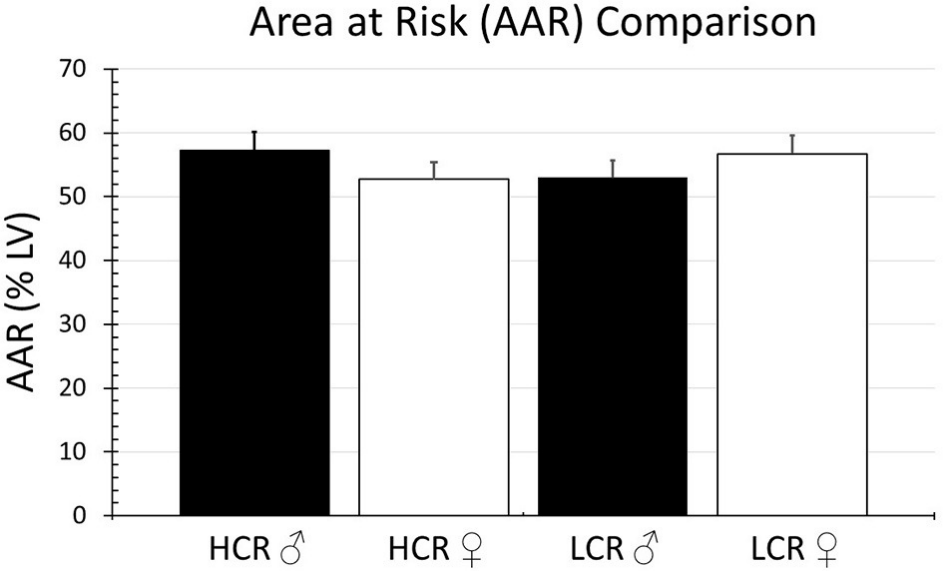
Comparison of the percentage of the LV at risk for ischemia (AAR) in the regional ischemia model. The model was reproducible, and the amount myocardium rendered ischemic by coronary occlusion was comparable in all groups.

In the regional ischemia model, ligature placement was consistent and there were no significant differences in the area at risk among the groups (HCR males = 57.3% ± 4.0, LCR males = 53.0% ± 3.7, HCR females = 52.8% ± 3.2, LCR females = 56.7% ± 3.6) (Figure 1).

### Myocardial Infarct Size

**Figure 2:**
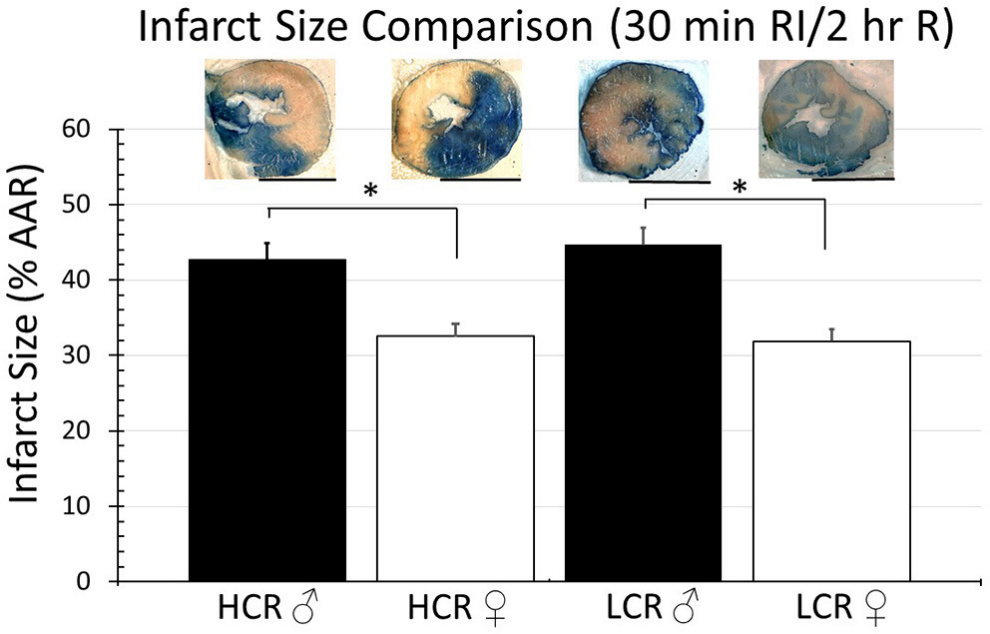
Comparison of the average infarction size normalized to area at risk (%AAR) between HCR male/female and LCR male/female rats after 30 min ischemia and 2 h reperfusion using an *in situ* regional ischemia (RI) model. Scale bars in images indicate 5 mm. Significant differences were noted between sexes within each phenotype, but not between phenotypes for either sex (*p < 0.05 for the comparison indicated).

**Figure 3:**
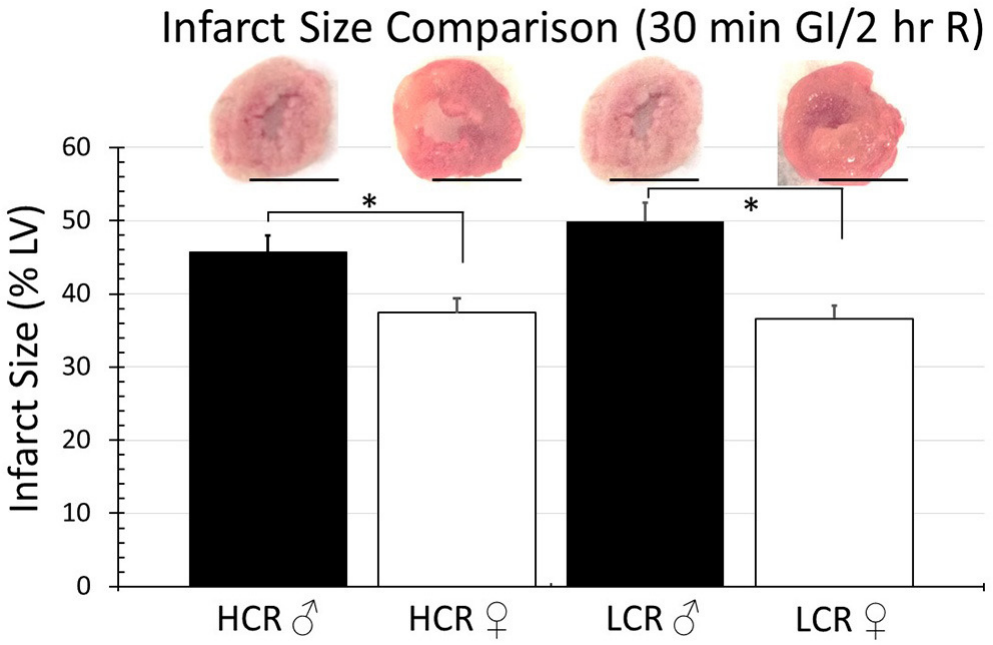
Comparison of the average infarction size normalized to the whole heart between HCR male/female and LCR male/female rats after 30 min ischemia and 2 h reperfusion using an *ex vivo* buffer-perfused global ischemia (GI) model. Scale bars in images indicate 5 mm. Significant differences were noted between sexes within each phenotype, but not between phenotypes for either sex (*p < 0.05 for the comparison indicated).

Thirty minutes of regional ischemia followed by 2 h of reperfusion generated no differences in infarct size between HCR and LCR. Infarct size was consistently smaller in female compared to male rats within each phenotype, but not between phenotypes (HCR males = 42.7% ± 4.3, LCR males = 44.7%, HCR females = 32.6% ± 3.2, LCR females = 31.9% ± 3.6) (Figure 2). Similar results also were seen in the global ischemia model (HCR males = 45.7% ± 4.4, LCR males = 49.9% ± 4.7, HCR females = 37.5% ± 3.6, LCR females = 36.6% ± 3.9) (Figure 3).

**Figure 4:**
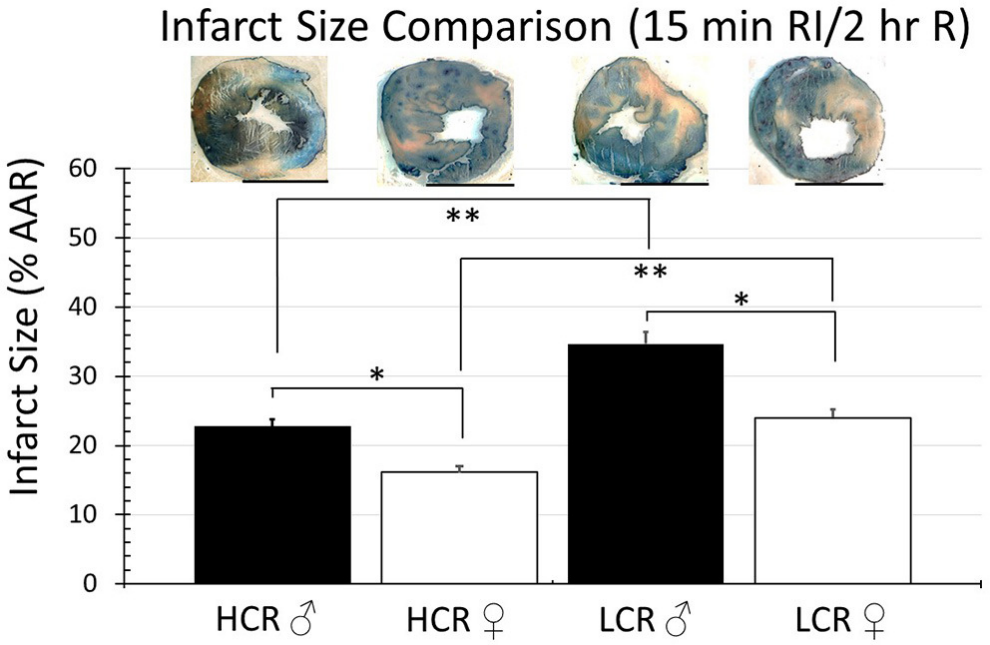
Comparison of the average infarction size normalized to area at risk (%AAR) between HCR male/female and LCR male/female rats after 15 min ischemia and 2 h reperfusion using an *in situ* regional ischemia (RI) model. Infarct sizes were smaller for all groups compared to 30 min regional ischemia (Figure 2). Scale bars in images indicate 5 mm. Significant differences were noted between sexes within each phenotype, and also between phenotypes for each sex, with LCRS having larger average infarctions compared to HCRs (*p < 0.05 for the comparison indicated between sexes within phenotype; **p < 0.05 for the comparison indicated between phenotype within sex).

**Figure 5:**
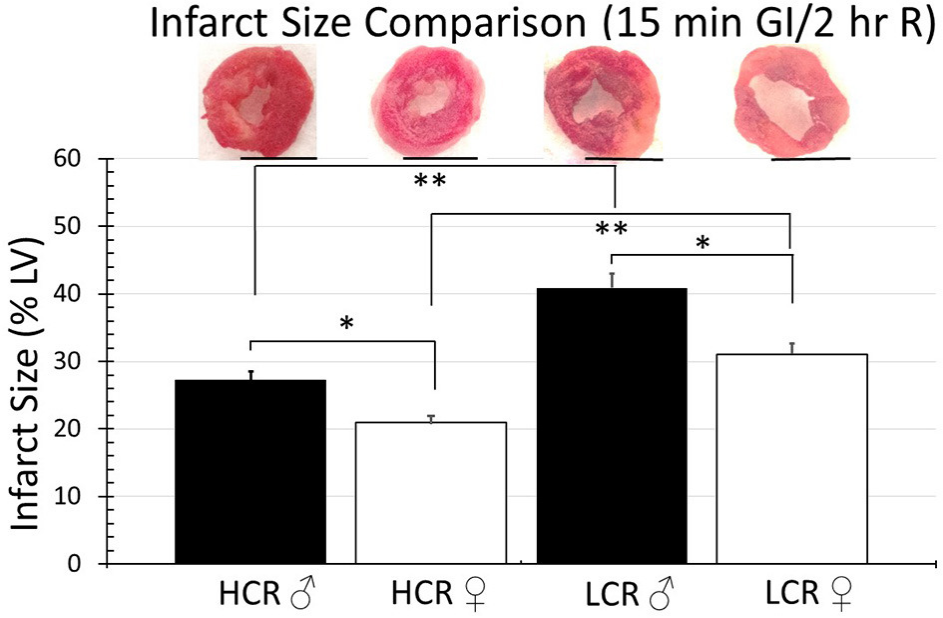
Comparison of the average infarction size normalized to the whole heart between HCR male/female and LCR male/female rats after 15 min ischemia and 2 h reperfusion using an *ex vivo* buffer-perfused global ischemia (GI) model. Infarct sizes were smaller for all groups compared to 30 min global ischemia (Figure 3). Scale bars in images indicate 5 mm. Significant differences were noted between sexes within each phenotype, and also between phenotypes for each sex, with LCRS having larger average infarctions compared to HCRs (*p < 0.05 for the comparison indicated between sexes within phenotype; **p < 0.05 for the comparison indicated between phenotype within sex).

Thirty minute occlusion is the most common model in rat, but it is intended to produce substantial injury (near 50% of the risk area in regional ischemia; 40–50% of LC in global ischemia) in order to detect a meaningful effect when testing the efficacy of injury reducing interventions. To determine whether the insult was simply too severe and potentially overwhelmed any innate differences between phenotype, the studies were repeated but with occlusive time reduced to 15 min, again followed by 2 h of reperfusion. Under these circumstances, there was an expected overall reduction in infarction size in all groups, regardless of sex or phenotype, compared to the 30 min ischemia results. However, HCRs, both male and female, showed a larger decrease than the LCRs, and a reduction in infarct size by phenotype consistent with relative cardioprotection in HCRs that was phenotype, and sex specific (HCR males = 22.7% ± 2.9, LCR males = 34.7% ± 3.3, HCR females = 16.2% ± 2.0, LCR females = 24.0% ± 2.1) (Figure 4). Similar results also were seen in the global ischemia model (HCR males = 27.2% ± 2.4, LCR males = 40.9% ± 3.9, HCR females = 20.8% ± 2.0, LCR females = 31.1% ± 2.9) (Figure 5).

**Figure 6:**
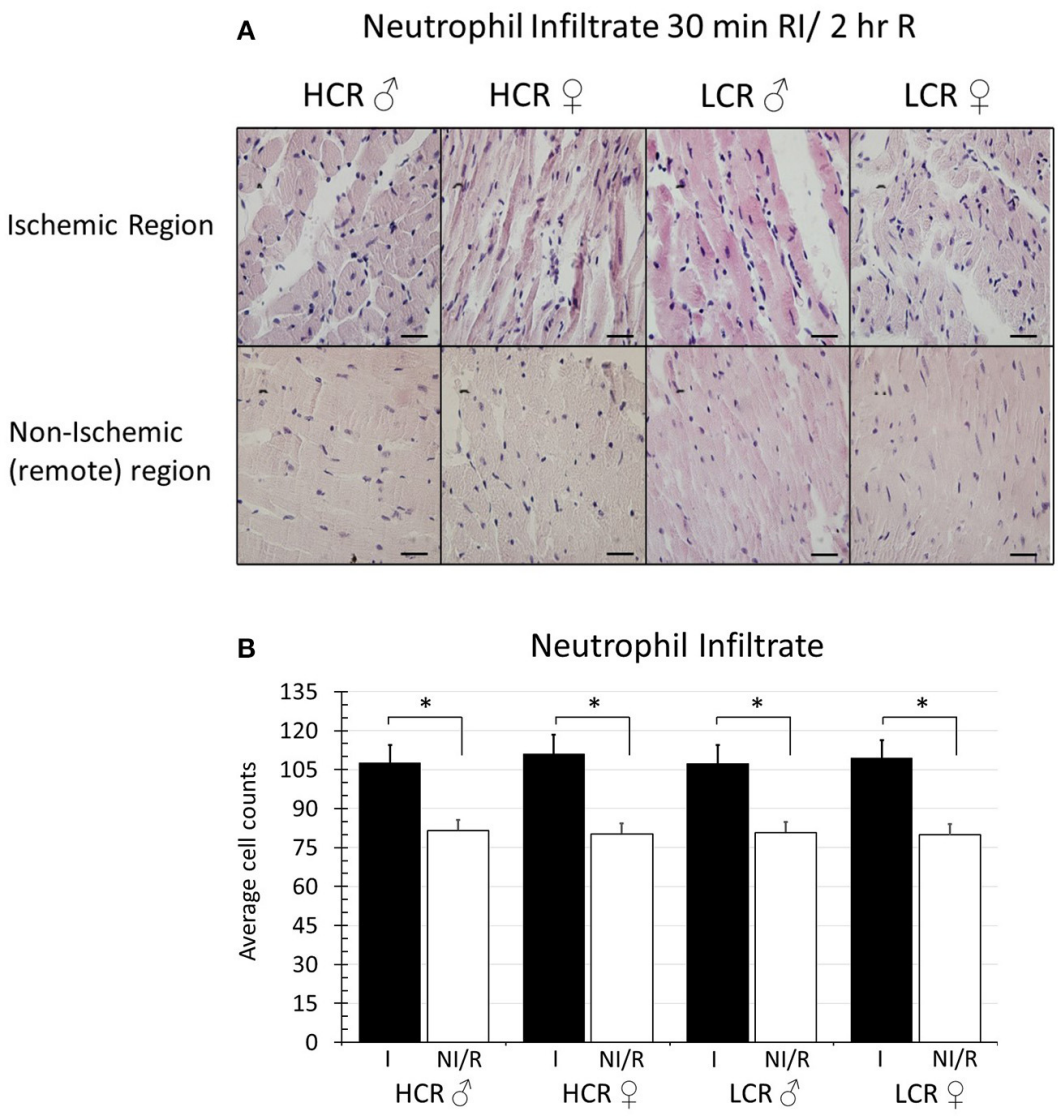
**(A)** Sample histologic sections showing relative myocardial neutrophil infiltration after 30 min *in situ* regional ischemia (RI) and 2 h reperfusion by sex and by phenotype in samples from the ischemic region, and from non-ischemic, remote regions (right ventricle) from the same hearts (H&E staining, 60X). Scale bars in images represent 200 um. **(B)** Quantification of neutrophil infiltration (cell counts). Data represents average of 5 fields in each section, and 5 sections for each animal. Ischemic regions (I) showed higher levels of infiltration compared to non-ischemic remote (NI/R) regions, but the increased levels of infiltration were not different between sexes within either phenotype, and were not different between phenotypes for either sex (*p < 0.05 for the comparison indicated).

Histologic assessment of neutrophil infiltration in tissue sections from regional ischemia groups revealed a generalized increase in infiltration in the ischemic region of about 20% above remote regions from the same heart (Figure 6A). However, the level of infiltrate was not different between phenotypes, and contrary to infarct size, also was not different between male and female in either phenotype (HCR M, Ischemic 107.5 ± 9.2, Remote 81.6 ± 5.8; HCR F, Ischemic 111.1 ± 9.7, Remote 80.3 ± 5.5; LCR M, Ischemic 107.4 ± 7.9, Remote 80.8 ± 6.3; LCR F, Ischemic 109.3 ± 9.8, Remote 79.9 ± 5.4) (Figure 6B). There also was no difference between 30 min ischemia and 15 min ischemia (data not shown), suggesting that infiltrate is driven more by reperfusion than by ischemia.

**Figure 7:**
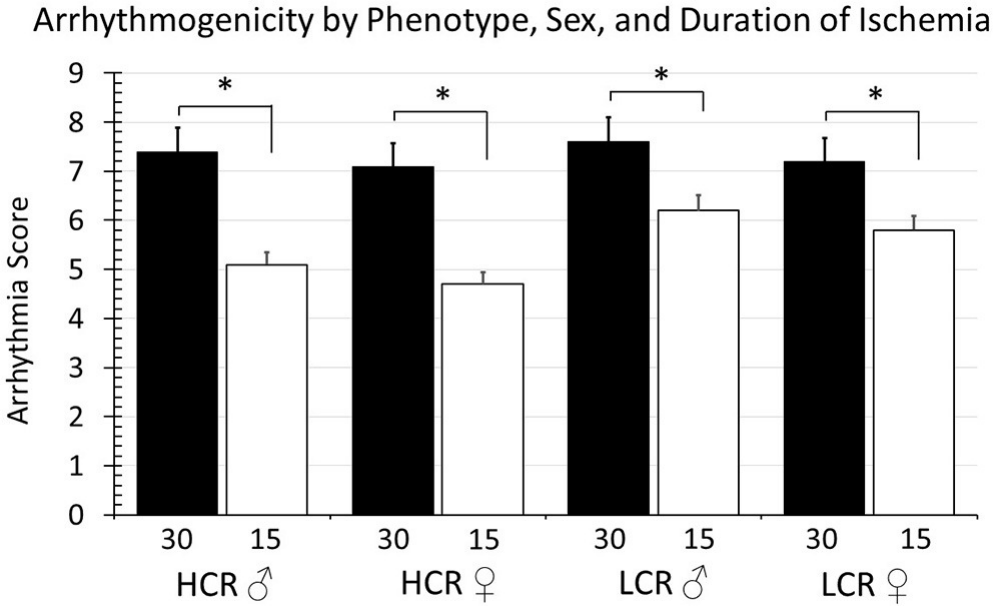
Summary of arrhythmia scores according to duration of ischemia (30 vs. 15 min), strain (HCR vs. LCR), and sex (males vs. female). There were significant differences in the severity of arrhythmia (*p < 0.05 for the comparison indicated), with 30 min ischemic episode producing consistently higher arrhythmia scores than 15 min events, however there were not significant differences between strain or between sexes with strains.

The arrhythmia scores in the global ischemia cohorts are summarized in Figure 7. The incidence of reperfusion arrhythmia was higher with 30 min ischemia vs. 15 min in all groups. There were not differences within phenotypes between male and female animals, and there were not differences within sex when comparing across HCR and LCR strains.

A summary of changes in left ventricular developed pressure (LVDP, mm Hg), and the positive and negative rate of pressures change (+dP/dt and –dP/dt mm Hg/s, respectively), is provided in Table 1. Functional performance indices showed substantial and significant decreases with ischemia compared to baseline for both ischemic times, but 30 min of ischemia did not further decrease the function, or alternatively, shortening ischemia to 15 min was not enough to significantly recover the function, even though tissue injury was reduced (infarct size, Figures 3, 5), suggesting that functional recovery is more complex than simple tissue salvage. There were no differences in these results between LCR and HCR strains comparing the same sex, or within either strain in male female comparisons.

**Table 1:** Summary of functional variables in global ischemia groups, by Phenotype, Sex, and duration of global ischemia.

	**HCR ♂**		**HCR ♀**		**LCR ♂**		**LCR ♀**	
**Variable**	**30I/120R**	**15I/120R**	**30I/120R**	**15I/120R**	**30I/120R**	**15I/120R**	**30I/120R**	**15I/120R**
LVDP-B	122 ± 12	118 ± 11	123 ± 11	119 ± 14	124 ± 10	119 ± 11	118 ± 13	119 ± 12
LVDP-R	22 ± 3	28 ± 4	26 ± 3	33 ± 3	24 ± 3	27 ± 7	27 ± 2	31 ± 3
+dP/dt B	4,332 ± 222	4,458 ± 355	3,997 ± 402	4,223 ± 405	3,987 ± 441	4,133 ± 367	3,994 ± 422	4,511 ± 333
+dP/dt R	721 ± 126	755 ± 155	758 ± 147	802 ± 112	776 ± 131	780 ± 142	818 ± 152	827 ± 147
–dP/dt B	2,791 ± 225	2,595 ± 302	2,666 ± 378	2,805 ± 314	2,746 ± 387	2,656 ± 379	2,681 ± 396	3,648 ± 336
–dP/dt R	450 ± 57	490 ± 58	518 ± 59	525 ± 62	455 ± 67	489 ± 64	505 ± 58	515 ± 63

*30I/120R, 30 minutes global ischemia and 2 hours reperfusion; 15I/120R, 15 minutes global ischemia and 2 hours reperfusion; LVDB, LV developed pressure; B, baseline; R, reperfusion; +dP/dt, maximum rate of pressure development (mm Hg/sec); –dP/dt, maximum rate of pressure fall (mm Hg/sec)*.

## Discussion

In this study, using two different models of ischemia-reperfusion injury, it is clear that increased intrinsic aerobic capacity phenotype (HCR) did confer a level of cardioprotection relative to the reduced/low capacity phenotype (LCR) (Figures 4, 5). The finding is consistent with a more general characterization of the HCR as “disease/injury resistant” phenotype, and the LCR as a “disease/injury prone” phenotype (25–27). Both phenotypes demonstrated comparable relative injury reduction in females relative to males, suggesting that the factors responsible for determining intrinsic aerobic capacity are not sex dependent, but that the responses determining response to cardiac ischemic injury are. The finding that similar results were seen in both intact regional ischemia models and in buffer perfused ischemia models *ex vivo* tends to suggest that the factors responsible for the differences in phenotype had more to do with inherent coronary and cardiac tissue characteristics than with any differences in circulating blood factors, or autonomic influences.

Noteworthy however, is that the cardioprotection associated with the HCR phenotype was lost with longer durations of ischemia. Extending the ischemic time from 15 to 30 min eliminated the difference in infarct size between the phenotypes (Figures 2, 3). The sex difference within each phenotype remained. Relatively speaking, the difference in infarct size between 15 and 30 min was actually larger in the HCR than in the LCR, consistent with an accelerated rate of injury once the threshold for protection had been exceeded. The reperfusion periods were the same with both time periods. Neutrophil infiltration is a common hallmark associated with reperfusion injury and was not different between the phenotypes, suggesting the possibility that the loss of relative cardioprotection was more likely due to overwhelming intrinsic cardiac or coronary features during ischemia, rather than reperfusion.

The HCR phenotype has been associated with increased longevity generally (32), which means it might also be possible that there is less benefit from the phenotype in an acute injury model such as the one used in this study, than in a more chronic, progressive disease setting such as high fat feeding (18, 33). It is also possible that the effect seen here is tissue dependent, but others have reported no difference between phenotypes in survival from multisystem stress in a model of hemorrhagic shock (34). We also did not complete any longer-term outcomes following the ischemia reperfusion injury. It is possible that long term results in myocardial healing and progression to heart failure might have been different between phenotypes, despite similarities in initial injury (35, 36). Certainly, previous studies using this model have shown that the immune response is exaggerated in LCR (25–27), and particularly TNF-α (37) as well as interleukin-10 (38), all of which could alter the progression of post-ischemic remodeling. It also is important to note that lack of ischemic protection despite interventions such as exercise and diet control have been reported in humans as well when injury is sufficiently severe (39, 40).

Gender has been recognized as an important factor in determining the risk for cardiovascular diseases (23) and cardioprotective effects of sex hormones have been reported in both experimental and clinical studies (41). Moreover, gender differences have been well-established in models of ischemia reperfusion injury (23, 24). Bae and Zhang observed significantly less myocardial injury in female vs. male hearts following 25 min of ischemia and 2 h of reperfusion in Langendorff-perfused rat hearts (42). The current results suggest that gender effects on cardiac ischemic tolerance are preserved despite selection for intrinsic aerobic capacity. While there is much still to be determined, it is becoming increasingly clear that many of the sex related differences in outcomes from cardiovascular diseases could be related to sexual dimorphism at the mitochondrial level. Estrogen has a substantial influence on mitochondrial gene expression, which in turns influences biogenesis, apoptosis, energy production, calcium handling and ROS production (35).

In humans there is a strong association between active exercise and cardiovascular protection (43). Regular physical exercise has been shown effective in the secondary prevention of cardiovascular disease and is effective in attenuating the risk of premature death among men and women (44). In other rat models, it has been demonstrated that acute exercise training can induce cardioprotection that results in reduced infarctions following ischemia–reperfusion injuries (45, 46). However, there can be quite notable strain dependent differences in response to a wide range of physiological challenges (47). Humans are not entirely well-modeled by inbred animal strains. The HCR and LCR phenotypes were developed by phenotypic selection using an outbred background, as a model to better assess the genetic components of aerobic capacity (25–27). The HCR/LCR model has inherited differences in aerobic capacity without prior training. Wisloff et al. found that the LCR phenotype scored high on cardiovascular risk factors and the HCR score high for health factors (15) while Lujan et al. demonstrated that the LCR phenotype demonstrated increased ischemia-reperfusion–mediated ventricular tachyarrhythmias and the HCR phenotype decreased susceptibility to tachyarrhythmias following short duration, non-infarction ischemia (48).

One interesting hypothesis is that the higher level of intrinsic aerobic capacity creates relatively less opportunity to “pre-condition” the heart, if in fact the phenotype is inherently less vulnerable to demand induced ischemia (31, 42, 49). Similarly, the LCRS might exceed a “pre-conditioning” threshold more easily. If so, it could mean that active exercise protocols intended to improve cardiovascular health would be more effective if first “titrated” against intrinsic capacity.

It is also clear from subsequent studies that the adaptive/induced response to a training regimen is genetically determined independently from the intrinsic aerobic capacity (50). Animals characterized as HCR or LCR appear to have similar capacity to improve (or not) when exposed to an active exercise regimen. There has been the suggestion that active exercise might “rescue” the LCR phenotype (51), but the fact that the factors governing the response to exercise training and the intrinsic exercise capacity seem to sort differently genetically (33, 42, 52, 53) would suggest that might not be the case. What is clear is that the availability of the HCR and LCR phenotype for the first time provides the opportunity for much better insight into the dynamic interaction between the intrinsic capacity for exercise, and the dynamic response to an active exercise regimen.

Consistent with our findings suggesting an intrinsic tissue character to the HCR/LCR phenotype effect, and the possibility that there might be tissue specific variation, several studies have suggested that a major component of the differences in intrinsic capacity might be metabolically driven (50, 51, 53–57). Noland et al. found changes in metabolic FFA utilization was affected by phenotype and was more pronounced in skeletal muscle while cardiac tissue did not demonstrate these differences even with high fat feeding (18).

In summary, there appears to be a cardioprotective benefit associated with inborn aerobic capacity. The relative cardioprotection can be overwhelmed if the ischemic stress sufficiently severe. Higher intrinsic capacity was associated with more rapid infarct expansion, once the protective threshold was exceeded. The relative impact of intrinsic aerobic capacity was not dependent on sex, with females showing less tissue injury regardless of phenotype or duration of ischemia compared to the males. The mechanisms for the phenotypic differences in ischemic tolerance appear to be intrinsic to the tissue, and more related to ischemia than reperfusion associated events.

## Data Availability Statement

The raw data supporting the conclusions of this article will be made available by the authors, without undue reservation.

## Ethics Statement

The animal study was reviewed and approved by Institutional Animal Care and Use Committee (IACUC) East Carolina University.

## Author Contributions

RML conceived experiments, analyzed data, wrote, and reviewed manuscript. MA, MZ, and LCK participated in data collection, analysis, and manuscript preparation. LGK and SB provided input on experimental design and model traits. LGK also reviewed data with RML. All authors contributed to the article and approved the submitted version.

## Conflict of Interest

The authors declare that the research was conducted in the absence of any commercial or financial relationships that could be construed as a potential conflict of interest.

## Publisher's Note

All claims expressed in this article are solely those of the authors and do not necessarily represent those of their affiliated organizations, or those of the publisher, the editors and the reviewers. Any product that may be evaluated in this article, or claim that may be made by its manufacturer, is not guaranteed or endorsed by the publisher.
